# Challenges and opportunities in technologies and methods for lncRNA structure determination

**DOI:** 10.1186/s13578-025-01470-2

**Published:** 2025-10-02

**Authors:** Maximilia Frazao De Souza Degenhardt, Yun-Xing Wang

**Affiliations:** Protein-Nucleic Acid Interaction Section, Center for Structural Biology, National Cancer Institute, National Institutes of Health, Frederick, MD 21702 USA

**Keywords:** lncRNA, Conformational dynamics, Conformational heterogeneity and flexibility, Single snapshot structure, Signal averaging, Ensemble of conformers, Conformational space

## Abstract

Long non-coding RNAs (lncRNAs) play pivotal roles in diverse cellular processes ranging from gene regulation and chromatin remodeling to RNA stability and epigenetic modifications. Despite the identification of approximately 95,000 lncRNA genes in humans, our understanding of their structure–function relationships remains very limited. This review examines the current state of lncRNA structure determination. We briefly discuss the advantages and limitations of experimental approaches—including chemical probing methods such as SHAPE and DMS—as well as the challenges inherent to computational predictions, particularly given RNA's dynamic nature, structural heterogeneity, and the energy degeneracy of its building blocks. The review also highlights the difficulties in predicting long-range interactions, including pseudoknots, which are essential for global folding of large RNAs, and discusses how elevated, nonphysiologically Mg^2^⁺ concentrations used in many experiments can distort our perception of native RNA conformations. Recent advances in cryo-electron microscopy and atomic force microscopy, coupled with machine learning algorithms, offer promising strategies to capture the realistic conformational landscapes of RNAs, including lncRNAs, under near-physiological conditions. These advances have the potential to redefine our understanding of lncRNA architectures, their structural dynamics, and how they influence cellular functions, ultimately informing future directions of lncRNA research and opening new frontiers such as structure-based drug discovery and therapeutic interventions targeting lncRNAs.

## Background

**Fig. 1:**
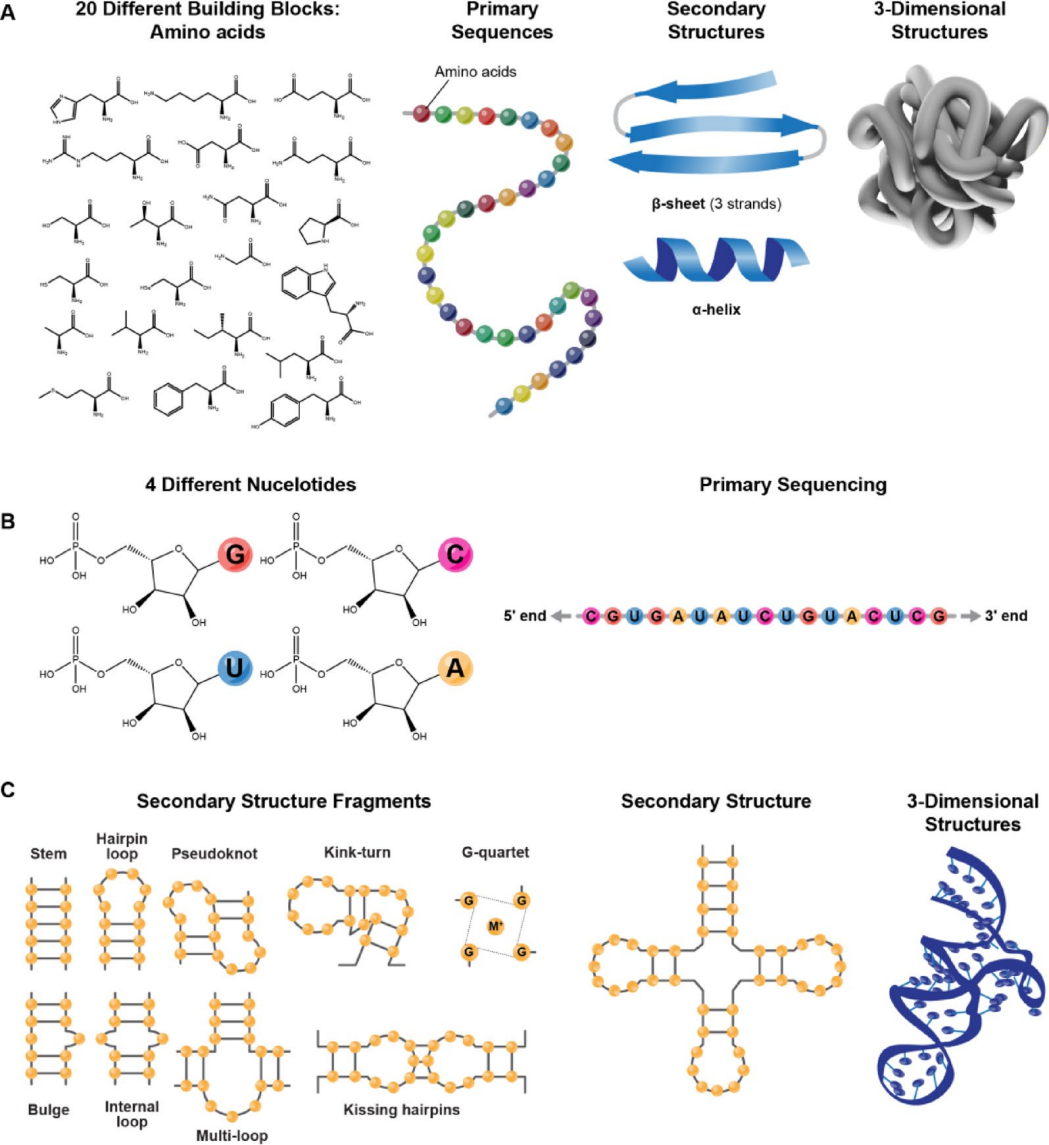
Comparison between protein and RNA: building blocks drawn in chemical structures, sequences with beads representing residues, secondary structures in cartoons, and 3D structures in cartoons. Note that twenty different types of amino acids are made up of proteins (**A**), compared to only four types of nucleic acids for RNA (**B**). On the other hand, there are more varieties of structural elements in RNA (**C**) than in protein (**A**)

Compared to proteins, RNA presents unique challenges to structural biologists due to its unique chemical composition, intricate structures, and the inherently anionic nature of its building blocks (Fig. 1). As a polyanionic polymer, RNA folds into complex three-dimensional (3D) structures through a delicate balance of stabilizing interactions, such as base stacking and hydrogen bonding, and repulsive forces like Coulomb repulsion among phosphate groups. Each nucleotide contributes 11 torsion angles with considerable rotational freedom, which are the origin of the overall flexibility of an RNA molecule. Consequently, longer RNA chains possess more torsional degrees of freedom, leading to increased conformational variability. The flexibility of an RNA is vividly illustrated by the extensive flexibility observed in a small 29-nucleotide RNA hairpin with an internal bulge [1]. One can readily extrapolate this phenomenon to much larger RNA molecules, including long non-coding RNAs (lncRNAs), which are the focus of this review by highlighting both the challenges in structural determination posed by RNA’s conformational heterogeneity and the opportunities it presents for advancing our understanding of RNA structure and function.

The X-inactive specific transcript (XIST), responsible for X-chromosome inactivation in female mammals, was among the earliest long non-coding RNAs (lncRNAs) identified in 1991 [2, 3]. Subsequent research has currently cataloged approximately 95,000 lncRNA genes in the human genome [4], a number exceeding the total number of protein-coding genes (< 20,000) [5] by more than fourfold. Of these human-expressed lncRNAs, approximately 1900 are conserved across primate species [6]. lncRNAs exert a broad range of regulatory functions, influencing gene expression [7, 8], chromatin remodeling⁷, RNA stability and degradation [9–11], RNA decoy and scaffolding mechanisms [12], cell cycle regulation and apoptosis [13, 14], nuclear organization and dynamics [8, 15], and cellular development and differentiation [7, 16, 17]. These examples underscore the crucial roles of lncRNAs in biological systems. Despite substantial progress in lncRNA research, the field remains an emergent frontier within RNA biology, with significant knowledge gaps persisting. In particular, the structure–function relationships and the structural underpinnings of lncRNA regulatory mechanisms remain elusive, posing considerable challenges to elucidating their intricate roles and interactions in cellular processes. Several authoritative reviews have previously discussed lncRNA structure determination [18–20]. This short review focuses on the current challenges and emerging opportunities in lncRNA structure determination, in light of recent methodological and technological advancements and their implications [21–23].

### Secondary structure landscape

In addition to serving as an indicator for potential functions and activities, the secondary structures of lncRNAs and understanding them are foundational, as they inform the design of constructs for three-dimensional (3D) structure determination, and are essential for accurate 3D structure prediction using computational methods. Moreover, it reveals evolutionarily conserved and functionally significant structural motifs. Secondary structures are mapped using a number of experimental techniques, computational tools, or integrative approaches combining both. Experimental methodologies include in vitro SHAPE (Selective 2′-Hydroxyl Acylation analyzed by Primer Extension) [24, 25], in vivo SHAPE [26], DMS (Dimethyl Sulfate) Probing [27–29], Structure-Seq, SHAPE-Seq [24], and DMS-MaPseq [26]. These high-throughput hybrid methods couple chemical probing with next-generation sequencing to achieve nucleotide-resolution structural insights across the transcriptome, both in vitro and within living cells. As with all experimental approaches, these techniques are subject to inherent limitations. SHAPE-directed analyses of highly structured RNAs exhibit a false negative rate of 17%, a false discovery rate of 21%, and less than 50% confidence in certain regions, due to insufficient information content in SHAPE data, constraints in data processing and normalization, and flexibility in helical regions [30], as of note, this data maybe outdated, as no significant updates have been published in recent years. Errors may also arise from misinterpretations of accessibility data, predicated on assumptions of singular or limited conformations, whereas RNAs may exhibit heterogeneous folding or dynamically explore expansive conformational landscapes [21, 31].

Comparable to other RNA classes, lncRNAs comprise diverse structural elements—including helices, terminal and internal loops, junctions, pseudoknots, triplexes, and G-quadruplexes—that fold into distinct 3D architectures (Fig. 1). Post-transcriptional modifications could further diversify lncRNA conformations, modulating their interactions with binding partners [32, 33] and adding complexity to their structural–functional dynamics. Genome-wide secondary structure mapping has established that lncRNAs are highly structured, many of which are organized into domains of recurring structural motifs [34–36] with structural complexity comparable to well-structured RNAs such as RNase P RNA and introns.

Fewer than twenty lncRNAs from diverse organisms have had their secondary structures experimentally mapped [19, 37]. These include the steroid receptor RNA activator (SRA) [34], X-inactive Specific Transcript (XIST) [38, 39], Metastasis-Associated Lung Adenocarcinoma Transcript 1 (MALAT1) [40], Hox antisense intergenic RNA (HOTAIR) [36], Nuclear enriched abundant transcript 1 (NEAT1) [41], RNA on the X1 and X2 (ROX1/ROX2) [35], Braveheart [42], COOLAIR [43], SPRIGHTLY [44], SRA-like non-coding RNA [45], P21 [46], Maternally expressed gene 3 (MEG3) [47, 48], Polyadenylated nuclear RNA [49], NORAD#1–3 [50], CRYANO [51], RepA [52], and PAN [49]. These lncRNAs span a size range from 0.4 kb (COOLAIR [43] and SRA-like non-coding RNA [45]) to 17 kb (XIST [38, 39]). Genome-wide mapping indicates that lncRNAs are highly structured [34–36], with a structural complexity exceeding that of mRNAs but falling short of ribosomal RNAs [53–55]. Some mappings have also identified protein-RNA interaction regions [26, 36, 56] emphasizing the necessity of 3D structure determination for these interfaces. Pseudoknots, identified in SRA [45], XIST [38, 39] MEG3 [47] and HOTAIR [36], may be more prevalent in lncRNAs. Both experimental and theoretical evidence suggest pseudoknot formation is a critical determinant of RNA folding, stability, and function [57–59]. Pseudoknots are disproportionately enriched in functionally critical motifs across large catalytic RNAs, riboswitches, and viral regulatory elements [5, 58, 60, 61]. However, current experimental methods struggle to detect pseudoknots, which are distinctive features of long-range secondary structure interactions. In a pseudoknot, a loop from one part of the sequence forms base pairs with another distant region in the primary sequence. Current methods also face challenges in detecting other types of long-range interactions. This limitation primarily arises because these techniques rely on local signals, where the chemical environment is cleaner, with low signal overlap and minimal steric clashes.

Recently, AI models have emerged in RNA secondary structure prediction, aiming to reduce reliance on traditional thermodynamic models that focus primarily on minimizing free energy. Initial studies have shown significant improvements in overcoming some of the challenges faced by classic approaches, particularly in predicting more complex interactions, such as non-canonical base pairing [62–65]. However, these AI-based approaches still fall short when it comes to predicting the structures of long RNA sequences. Several factors may contribute to this limitation, including the relatively small datasets available for training the models, the potential inadequacy of certain AI architectures, or the inherent complexity and variability of RNA folding itself. Thus, experimental approaches are challenged by data from heterogeneous samples such in physiologically relevant low Mg^2+^ solutions or in vivo, while computational methods are limited by data scarcity, overfitting, and model complexity, despite recent progress in high-throughput sequencing coupled with probing with improved resolution. Combining both approaches might be a viable path forward [66].

Overall, there is a lack of evidence suggesting long-range interactions involving both 5’- and 3’-ends—common in large well-folded RNAs such as rRNA, group-I and II introns, and RNase P RNA. This may be due to limitations in current mapping technologies and computation algorithms in detecting such long-range interactions. On the other hand, it may suggest that lncRNAs predominantly consist of modular domains connected by single-stranded regions, analogous to beads on a string.

### Three-dimensional structures

Three-dimensional (3D) structural data are indispensable for elucidating the functions of biomacromolecules, including lncRNAs, as their biological roles are intricately related to their molecular architecture. The spatial arrangement of domains, structural elements, and critical residues governs lncRNA interactions with proteins, peptides, DNA, and other RNAs, underpinning their functional contributions to cellular processes. Such 3D insights are vital for delineating interaction interfaces, which are essential for understanding regulatory mechanisms and functional specificity in multicomponent systems. For instance, lncRNAs can act as scaffolds, orchestrating the assembly of histone modifiers and DNA methyltransferases at specific genomic loci for epigenetic regulation [67], with their spatial configurations and protein interactions dictating chromatin engagement. Additionally, lncRNAs undergo epigenetic modifications such as in m⁶A and m^5^C, thus conformational changes, which enhance or inhibit binding to epigenetic enzymes. The m⁶A modification of XIST, for example, enhances its interaction with chromatin remodelers during X-chromosome inactivation by adopting a compact conformation [68, 69]. Furthermore, 3D structural data are critical for structure-based drug discovery and development, revealing precise interaction interfaces and targetable regions essential for disrupting disease-associated lncRNA interactions with biomacromolecules or guiding antisense oligonucleotide design [70]. Potential targets include lncRNAs scaffolding epigenetic complexes, guiding DNA methylation [61] and histone modifications [69], or oncogenic lncRNA structural motifs in cancer. To date, only a very few high-resolution 3D structures of lncRNA structural elements, such as a triple helical motif in MALAT1 have experimentally been determined [71]. This novel structural element is believed to confer stability.

Relative to proteins and other RNA types, experimentally determined 3D structures of lncRNAs, or structural elements, remain scarce. Recent efforts employing low-resolution methods have yielded some progress. Chemical probing, covariant analysis, and UV-crosslink data of RepA suggest it comprises three independent folding domains [52] with tertiary long-range interactions, proposing an initial structural model of two interacting subdomains [52]. Braveheart lncRNA, analyzed via small-angle X-ray scattering (SAXS) and chemical probing, exhibits structured flexibility and compaction at elevated Mg^2^⁺ concentrations [72], undergoing structural remodeling upon binding the protein CNBP. Direct visualization techniques, such as atomic force microscopy combined with secondary structure chemical probing, have also been employed to characterize lncRNA 3D structures [47, 73, 74].

### Challenges and opportunities

#### Limitations of computational approaches

Both computational and experimental strategies have been applied for RNA structure modeling and determination. Computational efforts emerged nearly four years after the first 3D tRNA crystal structures were reported [75–77], with the initial RNA 3D structure prediction published in 1991 [78]. Recent advancements, particularly those incorporating machine learning and deep learning following successes in protein structure prediction [79–81], have significantly advanced the field forward, as detailed in comprehensive excellent reviews [82–87]. However, whether RNA structure prediction can achieve parity with protein prediction using analogous methodologies remains uncertain [86]. While 3D predictions for small RNAs (< 100 nucleotides) with reasonable accuracy are currently feasible, they lag far behind protein prediction capabilities [88–90]. Several factors caution against overly optimism for RNA 3D structure prediction using protein-inspired strategies: (1) a paucity of high-quality, non-redundant RNA structures limits deep learning applications; (2) unlike proteins, RNAs lack a clear sequence-to-3D structure correlation; (3) multiple conformations for a single RNA sequence, especially under physiological Mg^2^⁺ concentrations [21, 23], confound single-structure predictions; and 4) RNA structure databases fail to capture the full conformational space, often reflecting non-physiological Mg^2^⁺ concentrations (e.g., hundreds of mM) [91], skewing structural representations. These challenges guide the focus of this review on experimental approaches and their opportunities.

#### General challenges

The 3D structure of a full-length lncRNA has yet to be resolved. Their large sizes—often kilobases or tens of kilobases—render techniques like NMR spectroscopy impractical. Additionally, their conformational heterogeneity and flexibility challenge methods such as X-ray crystallography and cryo-electron microscopy (cryo-EM). To contextualize these difficulties, it is instructive to consider some fundamental properties of RNA molecules and their solution behaviors.

**Fig. 2:**
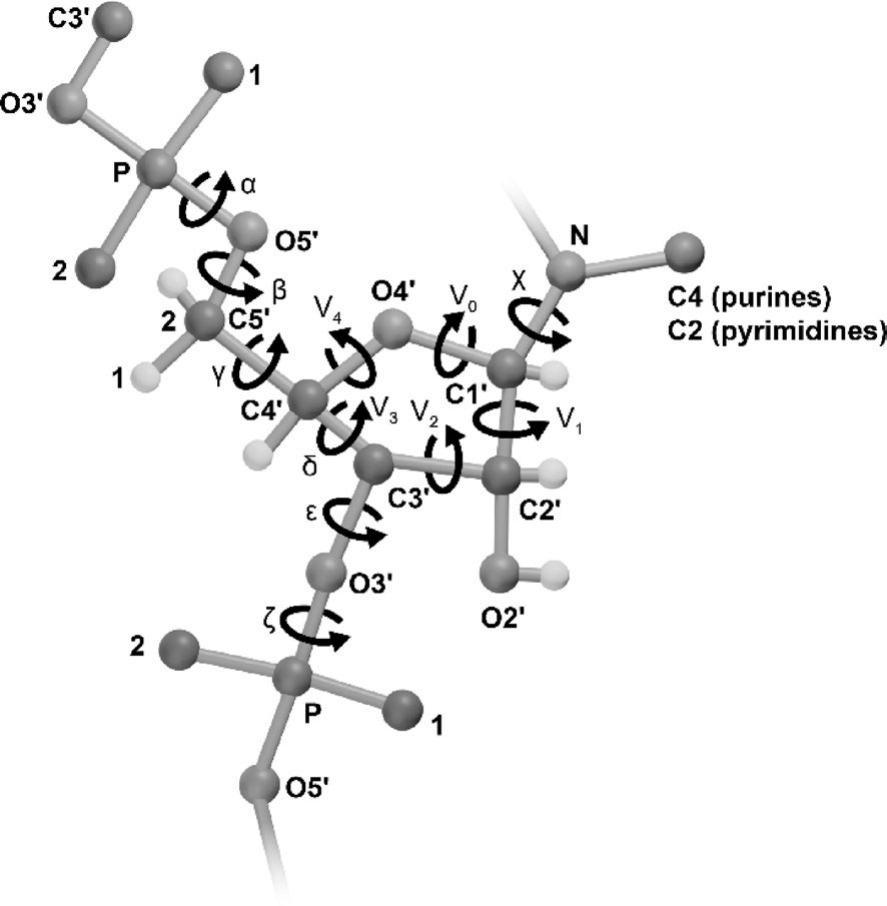
Torsion angles α, β, γ, δ, ε. And ζ along an RNA backbone, ν_0_ ν_1_ ν_2_ ν_3_ ν_4_ within a ribose ring, or χ around a glycosidic bond

**Fig. 3:**
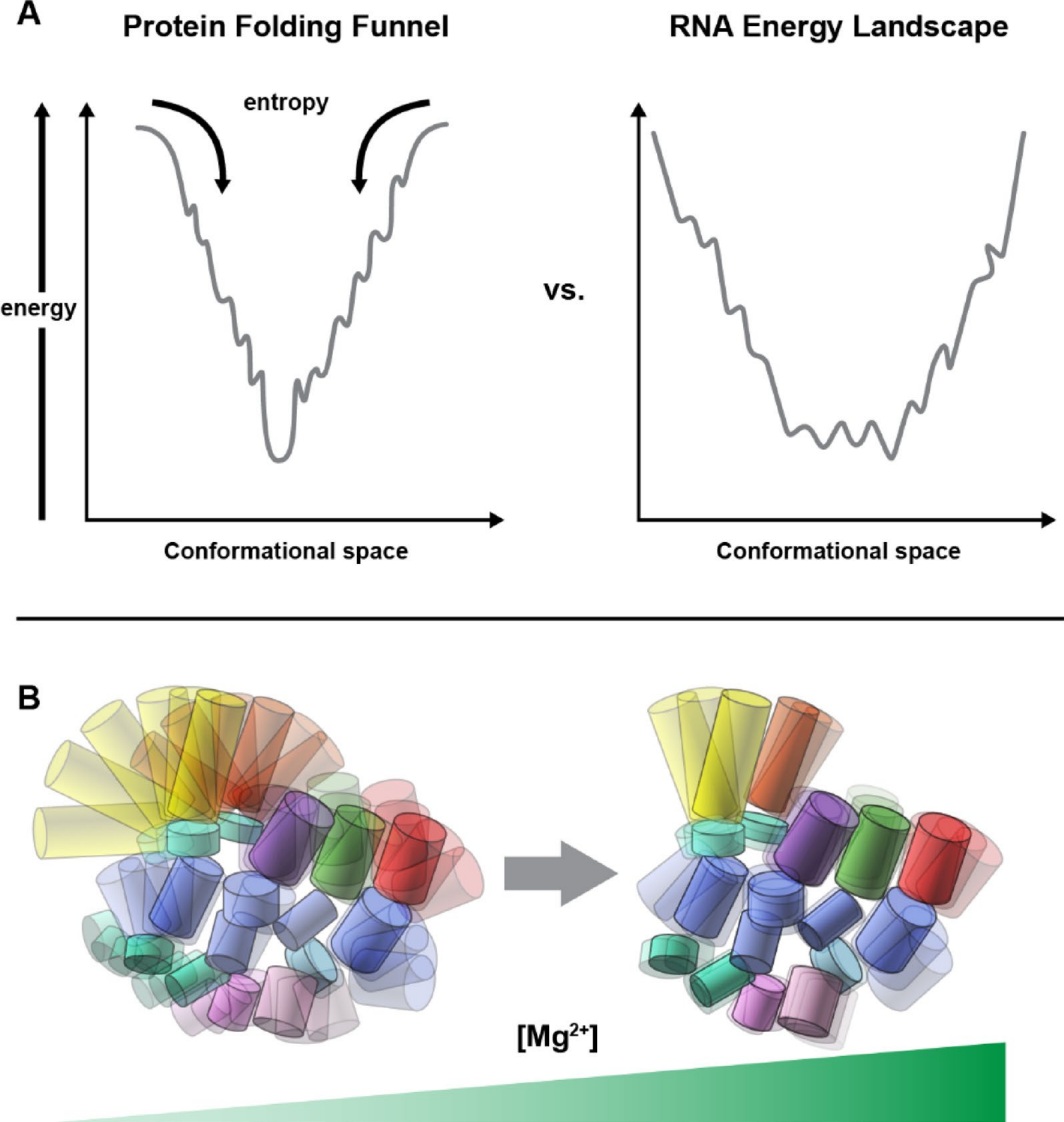
Energy landscapes of a well-folded protein and structured RNA (**A**), and Mg^2+^ dependent conformational flexibility and heterogeneity of structured RNA (**B**). Note that RNA has a rugged bottom of the energy well, whereas in protein, the energy landscape usually has a clear global minimum

Since the first protein structure was elucidated in 1958 [92], the paradigm of a single primary sequence folding into a defined secondary and tertiary structure driven by hydrophobic interactions and a limited set of secondary elements (e.g., α-helices, β-sheets) (Fig. 1) has dominated protein science. RNA, however, differs significantly in its chemical and structural properties, complicating both experimental and computational structure determination. RNA comprises four nucleotide types with a uniform sugar-phosphate backbone, differing only in bases (adenine, guanine, cytosine, uracil), contrasting with the twenty amino acids of proteins. This limited diversity results in structurally distinct base-pair arrangements with similar thermodynamic stability (degeneracy), as fewer nearest-neighbor combinations from four bases dictate free energy across configurations. Structural motifs—e.g., bulges, loops, junctions—formed from these bases exhibit minimal energy differences (e.g., U- or A-rich loops), hindering sequence-based 3D predictions based on stabilization energy. The sugar-phosphate backbone’s high rotational freedom across torsion angles contributes to conformational heterogeneity and flexibility (Fig. 2), lowering energy barriers between conformers and enabling dynamic sampling critical to function [21] (Fig. 3). Interconversion among coexisting conformers is quasi-isoenergetic, incurring minimal energy penalties [21]. Furthermore, the hydrophilic, negatively charged backbone influences folding and flexibility, responding to ionic conditions and adopting extended, dynamic conformations to minimize repulsive forces between phosphate groups. Altogether, these factors limit the utility of conventional methods reliant on signal averaging for the structure determination of lncRNA.

Beyond individual residues, RNA consists of a wide variety of structural elements that rely on canonical and non-canonical base pairing. Folded RNA structures are only metastable, balancing base-stacking, weak hydrogen bonds, and long-range interactions against Coulomb repulsion from negatively charged phosphate groups. The flexibility arising from high degrees of freedom throughout backbones and around glycosidic bonds render these structures susceptible to conformational fluctuations triggered by environmental shifts (e.g., temperature, ionic strength, pH) or induced fit upon binding ligands, proteins, DNA, or other RNAs.

In summary, RNA’s chemical composition and structural arrangements are the chemical and structural basis for its dynamic behavior and rugged energy landscape with numerous near-degenerate states coexisting. Small free-energy differences among conformations permit multiple coexisting conformers, interconverting via a quasi-isoenergetic process (QIEP) in physiologically relevant solution [21, 23], rendering RNAs—particularly under physiological Mg^2+^ concentrations—much more flexible and heterogeneous than proteins, or even under artificially elevated Mg^2+^ concentration. Such conformational flexibility is exemplified by one of the simplest RNA, a 29-nt hairpin of the HIV Tar RNA containing a two-residue internal bulge [93], or as large as the 417-nt RNase P RNA [21].

The pivotal role of Mg^2^⁺ in RNA structure and conformational dynamics, particularly its impact on RNA structure determination and the subsequent implications, warrants detailed discussion. RNA folding and conformational states are acutely sensitive to ionic environments, with Mg^2^⁺ as a critical divalent ion for folding and stabilization, alongside its contributions to catalysis and substrate binding. Mg^2^⁺ interacts with the phosphate groups of RNA, attenuating Coulombic electrostatic repulsion between adjacent negative charges and reinforcing the stable and compact structures. Specifically, Mg^2^⁺ is essential for sustaining metastable RNA structures by the neutralization of negative charges along the RNA backbone, thereby enabling the formation of stable and compact architectures. Physiological Mg^2^⁺ concentrations are approximately 1 mM [94, 95]. Subtle perturbations in Mg^2^⁺ concentration affect the conformational landscape of RNA, a sensitivity less pronounced in proteins. Consequently, RNA structure determination often relies on elevated Mg^2^⁺ levels, a requirement due in part to the intrinsically dynamic and heterogeneous nature of RNA structures. This necessity underpins the determination of most RNA structures under non-physiological Mg^2^⁺ concentrations, reaching levels as high as 500 mM [91].

Now we turn to the technical limitations inherent to current methodologies, mainly Nuclear Magnetic Resonance (NMR), X-ray crystallography, and cryo-electron microscopy (cryo-EM). NMR excels in elucidating the structure and dynamics of smaller RNA fragments but is not suitable for large, kilobase-scale RNAs such as lncRNAs. Conversely, crystallography and cryo-EM are constrained by the physics of detecting weak X-ray or electron signals. Crystallography necessitates Bragg coherent diffraction from millions of ordered lattices, even with the most advanced X-ray free-electron lasers (XFELs), while cryo-EM requires tens of thousands of particles for robust volume reconstruction. Consequently, both approaches depend on substantial conformational uniformity within samples. As highlighted earlier, RNA exhibits significant dynamism and heterogeneity—particularly at physiologically relevant Mg^2^⁺ concentrations—even in well-structured forms [21, 23]. Thus, these techniques are limited to examining relatively homogeneous samples stabilized at elevated Mg^2^⁺ concentrations. The structures, captured under such non-physiological conditions, offer mere snapshots of the most compact and stable conformers. It should be mentioned that these structures diverge considerably from the physiological native conformation landscape, despite frequent designation as the "native" state in contemporary literature. Fundamentally, they only represent static depictions that fall short of capturing the extensive conformational diversity of RNA in solution, because several pioneering studies have already revealed that RNA structures are far more dynamic and heterogeneous than what could be described by single conformers [96–99].

#### Challenges unique to lncRNA

**Fig. 4:**
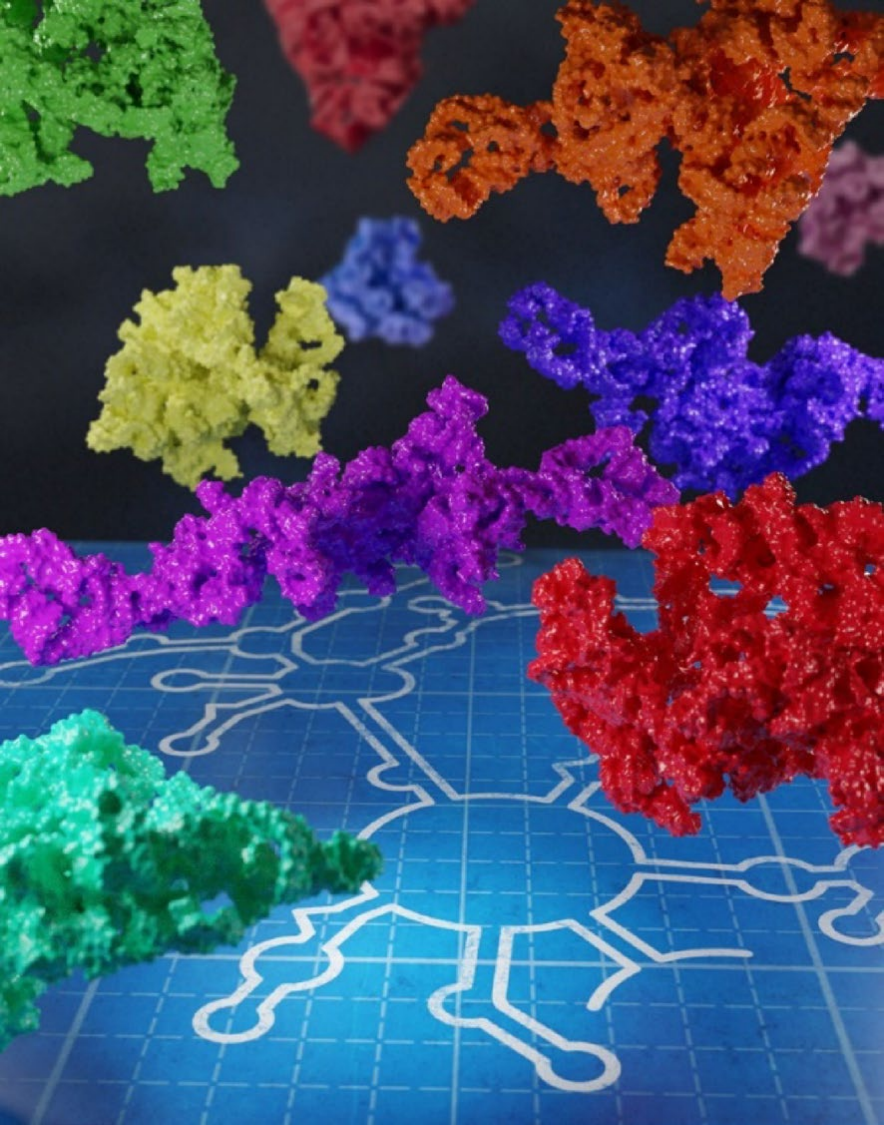
An artist's rendering of one sequence in the form of the secondary structure (white) of an RNA folding into multiple conformers (rendered in various colors and shapes). The structure models are artist-generated impressions representing different 3D conformers

In addressing long non-coding RNA (lncRNA) structure questions, the field of RNA structural biology faces several unique and significant challenges, and major advances are warranted to overcome these obstacles: (1). Complexity and large sizes; (2). Conformational heterogeneity; (3). Lack of experimental information about long-range interactions among various domains and modules; (4). Limited understanding of functional implications; (5). Low sequence conservation; The first three are directly relevant to structure determination. The foremost challenges—namely, their large size and conformational variability—limit the feasibility of the known conventional strategies and standard structural biology tools for structure determination. In particular, their propensity for adopting an ensemble of flexible conformations complicates the isolation of a representative state. Indeed, lncRNAs are posited to exist as dynamic and heterogeneous structural populations rather than very few discrete stably folded structures (Fig. 4). Overcoming these hurdles requires revolutionary new thinking and transformative advances in technologies and approaches capable of resolving the three-dimensional configurations of individual conformers, preferentially under physiologically relevant conditions regarding Mg^2^⁺ concentrations.

### New advances and hope

Cryo-electron microscopy (cryo-EM) has established itself as a powerful tool for elucidating the 3D structures of stably folded proteins and RNA molecules. Furthermore, recent advancements in cryo-EM data processing, particularly for conformationally dynamic systems, may possibly offer promising avenues for capturing high-resolution structural snapshots of heterogeneous conformers. Conformational heterogeneity may arise from continuous motions and discrete sampling. Cryo-EM analysis tools are undergoing progressive improvements, in particular, new developments using machine learning. A 3D model can be generated for a distinct and discrete sampling, using ab initio reconstruction, parsing the dataset into discrete conformational classes through iterative alignment and classification procedures [100]. For heterogeneous conformers with a consensus model, a 3D classification can be achieved, driving the analysis to sort different conformers within the data. Furthermore, an analysis can be performed by applying hybrid optimization algorithms based on maximum likelihood or Bayesian inference, unsupervised machine learning, as implemented in widely utilized software packages such as RELION [101] or cryoSPARC [102–104]. For continuous motions, one of the major challenges, methods like 3D Variability Analysis (3DVA) [105] or deep learning approaches such as CryoDRGN [106] and 3D Flexible.

Refinement (3DFlex) [107] (3DVA and 3D Flex are within cryoSPARC [102] package) enable the extraction of a continuous spectrum of conformational states from data. Nonetheless, the efficacy of these techniques has predominantly been demonstrated with large protein assemblies in the cases, wherein particle alignment and classification are relatively straightforward. The applicability of these methods to large RNA molecules, where almost every part of structures is flexible and heterogeneous and thus presents significant alignment challenges, remains to be conclusively demonstrated. Ultimately, a more profound challenge inherent to any signal-averaging-based approach is the prerequisite for a sufficiently populous subset of RNA conformers adopting identical conformations to enable robust volume reconstruction and become almost an intractable problem for large and complex RNA such as lncRNAs. This homogeneity in a populous subset of conformers may be achieved under elevated Mg^2+^ concentrations at expense of capturing a realistic conformational landscape.

High degrees of conformational heterogeneity of RNA require direct examination of individual molecules in solution.

The recent evolution of atomic force microscopy (AFM) enables such direct visualization with several clear advantages [108, 109]. It is a true single-molecule method and images are recorded in real space without the need for ensemble averaging, which is particularly useful for studying highly heterogeneous conformers. Unlike techniques like cryo-EM, which averages data from thousands of particles, AFM can be considered a 'shotgun' approach by visualizing individual molecules and capturing both discrete and continuous conformations without the need for averaging. AFM image data are recorded under near‐physiologically relevant conditions to capture the realistic solution behavior of RNAs as opposed to in elevated Mg^2+^ concentrations. **I**t detects both discrete and continuous conformational changes. AFM experiment requires only microliter volumes at nanomolar concentrations; and molecules can be observed without any manipulation, e.g., labeling, freezing, staining, or crystallization. Thus, the solution AFM method is well-suited for studying highly heterogeneous molecular systems under near-native conditions.

The usefulness of AFM was demonstrated by a recent study of the adenosylcobalamin riboswitch aptamer domain. The crystal structure of this RNA determined in 500 mM Mg^2+^ shows a compact structure [91]. In contrast, the direct visualization in an 1 mM Mg^2+^ solution shows this RNA adopts multiple conformational and architectural structures [23]. This diversity in conformer populations, as assessed through particle analysis, is substantiated by data from Small-Angle X-ray Scattering (SAXS) and isothermal titration calorimetry. The study also reveals that RNA multimerization arises from specific interactions—such as kissing loops and tetraloop-minor-groove docking—rather than non-specific forces like random base pairing or electrostatic effects, which one would otherwise suspect if no direct visual evidence were available. Nevertheless, determining the explicit structures of heterogeneous RNAs with accuracy estimates remains an unmet grand challenge given the low-resolution (~ 13 Å) nature of AFM particle images. This problem is solved in the next advance where individual RNA topological structures of conformers are determined from AFM particle images using unsupervised machine learning and deep neural networks implemented in a program HORNET [22]. Applying the high SNR of atomic force microscopy, this method is ideal for capturing structures of individual RNA molecules in distinct conformations and the accuracy of the structures is estimated to range from 3 to 6 Å, depending on the number of iterations of computation. Its capabilities were proven through analyses of the heterogeneous structures of RNase P RNA and the HIV-1 Rev response element (RRE) RNA. By bypassing the need for signal averaging—a limitation inherent in other techniques—this approach marks a breakthrough in elucidating the structures of large, flexible RNA molecules.

The HORNET’s capability to derive topological structures from AFM images of individual RNA conformers represents a transformative advancement in elucidating largely unexplored three-dimensional conformational space of RNA, far exceeding the limited static structures available in existing databases. The utility of HORNET was immediately demonstrated in the second paper of the back-to-back publications [21, 22], where the mapping of the full conformation space of RNase P RNA in 1 mM Mg^2+^ was reported. The *Geobacillus stearothermophilus* (*Gst*) RNase P RNA is 417-nt trans-acting ribozyme. Research by Lee et al. demonstrates that *Gst*-RNase P RNA exhibits pronounced flexibility and conformational heterogeneity, with its peripheral structural elements sampling a wide range of configurations with amplitudes of 20–60 Å across multiple directions. Remarkably, this extensive conformational sampling incurs minimal energetic cost, a phenomenon termed “quasi-isoenergetic” motion [21].

Despite this dynamic flexibility in more than 85% of its structure, *Gst*-RNase P RNA retains a conformationally invariant core of approximately 50 nucleotides. Structural comparisons with RNase P RNAs across all three kingdoms of life reveal that this invariable core structure remains strictly conserved [21], a finding consistent with its essential role in harboring the catalytic center and accessory protein binding surface. Further analysis uncovered significant correlations and anticorrelations between spatial flexibility and sequence conservation, indicating that the primary sequence intrinsically encodes the functional roles of both the structure and dynamics of critical regions. These insights collectively illuminate the structure-dynamics interplay that enables the RNA component of *Gst*-RNase P to execute both precise enzymatic activity and adaptability to diverse substrates.

Mapping the conformational space using HORNET demonstrates a new general approach to studying RNA structure and dynamics. Judging based on the secondary structure, 417-nt *Gst*-RNase P RNA is as complex as modules found in many lncRNAs, if not more complex. Since secondary structures of a number of lncRNAs have been experimentally determined, we expect that HORNET will be used to map the global and modular structures of lncRNAs in solution.

## Conclusions

The future of RNA structure determination is poised for significant advancement through the integration of cutting-edge technologies, such as machine learning and atomic force microscopy (AFM). Deep learning algorithms are rapidly enhancing their capacity to predict RNA structures with increased accuracy for relatively small RNA. However, it remains to be demonstrated if a purely computational approach could effectively address challenges posed by conformational heterogeneity and dynamic flexibility. The ability of these AI algorithms to process extensive datasets and synthesize multiple sources of information, including experimental data, holds significant promise for resolving the intricate details of RNA folding and intermolecular interactions that have historically eluded precise characterization. Nonetheless, relying exclusively on computational predictions without experimental restraints represents a largely unexplored frontier, particularly in deciphering the complex, degenerate conformational landscapes. As a result, AFM could be a unique, powerful tool for investigating RNA dynamics at the single-molecule level, delivering high-resolution, real-time measurements of conformational transitions and structural variability. Its capacity to visualize RNA molecules across diverse environments could make a comprehensive examination of their flexibility and adaptive responses possible, thereby yielding critical insights into their functional roles. As these technologies continue to evolve, they are poised to substantially enrich our understanding of the structural complexity of large and flexible RNAs such as lncRNAs, and ultimately inform more effective strategies for targeting RNA in therapeutic applications.

## Data Availability

Not applicable.
